# Mapping Mitotic Death: Functional Integration of Mitochondria, Spindle Assembly Checkpoint and Apoptosis

**DOI:** 10.3389/fcell.2018.00177

**Published:** 2019-01-10

**Authors:** Weimei Ruan, Hong Hwa Lim, Uttam Surana

**Affiliations:** ^1^Institute of Molecular and Cell Biology, Agency for Science, Technology and Research, Singapore, Singapore; ^2^Bioprocessing Technology Institute, Agency for Science, Technology and Research, Singapore, Singapore; ^3^Department of Pharmacology, National University of Singapore, Singapore, Singapore

**Keywords:** mitosis, mitotic checkpoint, mitochondria, apoptosis, mitotic death

## Abstract

Targeting the mitotic pathways of rapidly proliferating tumor cells has been an effective strategy in traditional cancer therapy. Chemotherapeutics such as taxanes and vinca alkaloids, which disrupt microtubule function, have enjoyed clinical success; however, the accompanying side effects, toxicity and multi drug resistance remain as serious concerns. The emerging classes of inhibitors targeting mitotic kinases and proteasome face their own set of challenges. It is hoped that elucidation of the regulatory interface between mitotic checkpoints, mitochondria and mitotic death will aid the development of more efficacious anti-mitotic agents and improved treatment protocols. The links between the spindle assembly checkpoint (SAC) and mitochondrial dynamics that control the progression of anti-mitotic agent-induced apoptosis have been under investigation for several years and the functional integration of these various signaling networks is now beginning to emerge. In this review, we highlight current research on the regulation of SAC, the death pathway and mitochondria with particular focus on their regulatory interconnections.

## Introduction

Mitosis (M phase) is characterized by a set of highly orchestrated cellular events. It is also the most dynamic phase of the cell division cycle when cells are exquisitely vulnerable to perturbations. Once a cell enters mitosis, a host of activities are initiated and completed in rapid succession: nuclear envelope breakdown, chromosomes condensation, Golgi fragmentation, mitochondria fission and partitioning of chromosome equally between the two poles of the cell by an intricate, microtubule-based dynamic structure known as the mitotic spindle (Jongsma et al., 2015). This is followed by cytokinesis that causes physical division of the progenitor cell resulting in the emergence of two genetically identical daughter cells. While cell cycle events are highly regulated and coordinated in normal cells, they are known to be deregulated in tumor cells, thus leading to rapid proliferation. Various therapeutic strategies have successfully targeted the M phase events to eliminate rapidly proliferating cancer cells (Rieder and Maiato, 2004; Manchado et al., 2012). The anti-microtubule agents such as taxanes and vinca alkaloids that perturb the mitotic spindle dynamics have long been employed as frontline chemotherapeutics to treat various cancers including metastatic breast, prostate, metastatic pancreatic, advanced ovarian, non-small cell lung, and lymphoma (Pignata et al., 2014; Blair and Deeks, 2015; Cookson et al., 2015; Delaloge et al., 2016; Lamar et al., 2016; Kang et al., 2018). Despite their clinical efficacy, several issues such as neurotoxicity, myeloid toxicity, and emergence of drug resistance pose significant challenges to the therapeutic use of these microtubule poisons (Dunton, 2002; Scripture et al., 2006; See et al., 2006; Gupta and Middleton, 2011). Extensive efforts have been directed at developing the second generation of anti-mitotic agents targeting different mitotic components. These new agents inhibit various mitotic regulators such as Plk1 (Polo-like kinase 1), Aurora kinase, Mps1(Monopolar spindle 1), and APC/C E3 proteasome (Chan et al., 2012; Mason et al., 2017). However, most have displayed poor efficacy and limited success compared to the classic microtubule poisons (Bavetsias and Linardopoulos, 2015; Gutteridge et al., 2016).

The ability of anti-mitotic agents to suppress tumor cell proliferation lies in their capacity to trigger cell death, induced either in mitosis during a prolonged mitotic arrest, or post mitotically following a premature exit from mitosis (Weaver and Bement, 2014; Dominguez-Brauer et al., 2015). The post-mitotic cell death may be therapeutically undesirable because of its incomplete penetrance in that the cells that escape post-mitotic death are aneuploid, can resume cell division cycle and may become genetically unstable, a feature generally thought to be associated with tumorigenesis and drug resistance (Dalton and Yang, 2009; Wang et al., 2017). A detailed picture of the signaling network through which mitotic death is modulated would therefore be desirable for a strategic therapeutic design that targets important nodes in this network.

An extended mitotic arrest, due to the activation of SAC or other causes, is a prerequisite for efficient induction of cell death during mitosis. SAC delays sister chromatid segregation and the mitotic exit in response to the impaired microtubule-kinetochore interaction and thus impedes mitotic progression. Cell death resulting from a prolonged period of mitotic arrest exhibits features similar to apoptotic cell death, such as mitochondrial membrane permeabilization, cytochrome c release and caspase activation (André et al., 2002; Yasuhira et al., 2016). The mechanism of SAC activation and its effects on the mitotic machinery are well established; however, little is known about the downstream targets of SAC (if any) that trigger mitotic cell death following an extended mitotic arrest. Although post-translational modifications of pro-apoptotic and anti-apoptotic proteins by mitotic kinases have been reported, it remains debatable whether such modifications are in themselves sufficient for initiating mitotic cell death. There are a number of studies exploring the potential feedback from apoptotic signaling to mitotic progression, emphasizing the link between upstream pro-apoptotic/anti-apoptotic proteins and the downstream caspase cascade in modulating cell death. However, mitochondria’s central role in the initiation of apoptosis in response to mitotic controls has not been explored extensively. Here, we examine the recent discoveries concerning the regulatory links among SAC, death pathway and mitochondria.

## The Spindle Assembly Checkpoint (SAC)

**FIGURE 1:**
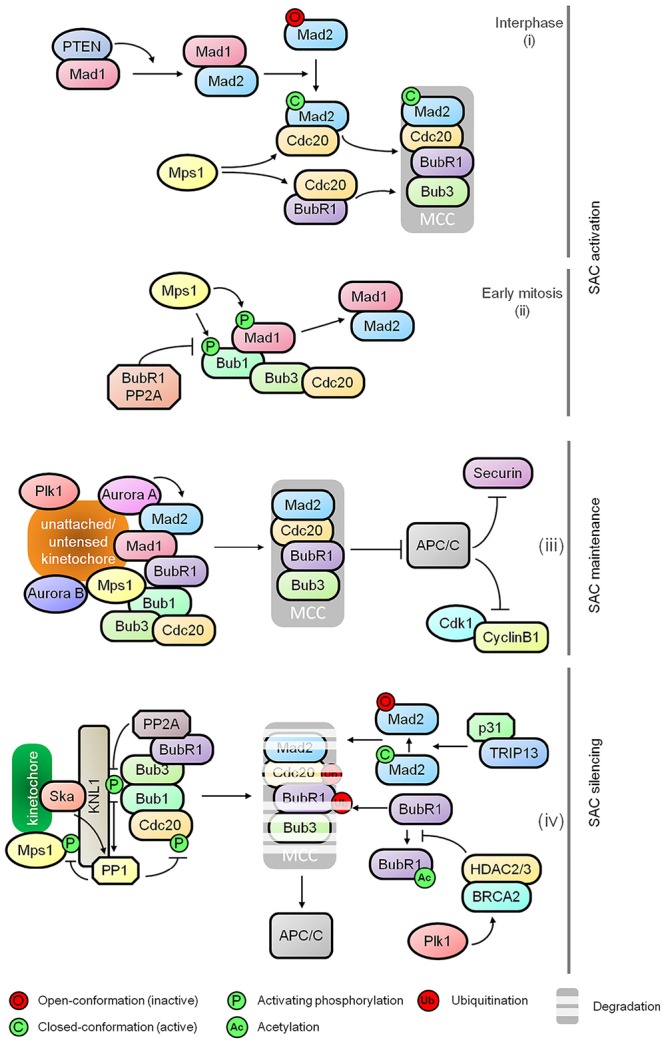
Regulatory dynamics of spindle assembly checkpoint (SAC). The schematic diagram shows the functional relationships between various effectors comprising the major control modules of the SAC: (i) SAC activation at nuclear pore during interphase, (ii) kinetochore independent regulation of Bub1 as the SAC activation timer during early mitosis, (iii) maintenance of the mitotic checkpoint complex (MCC), and (iv) destabilization of MCC and SAC silencing.

The SAC monitors the microtubule-kinetochore interactions during metaphase to anaphase transition. It is activated in the presence of unattached kinetochores and/or a lack of tension between sister kinetochores. The main effector of SAC is the mitotic checkpoint complex (MCC) which consists of BubR1 (Budding Uninhibited by Benzimidazole Related 1), Bub1 (Budding Uninhibited by Benzimidazole 1), Mad2 (Mitotic Arrest Deficient), and Cdc20 (Cell Division Cycle 20). The MCC effectively inhibits the activity of E3 ubiquitin ligase APC/C to prevent proteolytic degradation of securin and cyclin B1, the inhibitor of separase and the activator of Cdk1 (Cyclin-dependent Kinase 1), respectively (Figure 1). While Cyclin B1 stabilization results in sustained Cdk1 activity and the stabilization of securin that inhibits separase, thus preventing cohesin cleavage and sister chromatid separation.

Multiple kinases including Plk1, Mps1, Bub1 and Aurora B play critical roles in SAC signaling including kinetochore recruitment, activation and amplification of MCC as well as MCC-independent modulation of microtubule-kinetochore stability and APC/C inhibition. The control network underlying SAC activation has been described in detail in several excellent reviews (Lara-Gonzalez et al., 2012; Musacchio, 2015; Joglekar, 2016). Apart from the well-known attachment-dependent dynamics of SAC, recent studies provide further mechanistic insights into SAC activation during early onset of mitosis. It has been shown that MCC assembly is initiated at the nuclear pore during interphase by Mad1 mediated activation of Mad2. This requires PTEN for Mad1 dimerization and localization, and Mps1 mediated formation of the Cdc20-inhibitory complex with Mad2 and BubR1. It is suggestive of a SAC-priming event that generates an inhibitory signal for sister chromatid separation prior to the onset of mitosis before nuclear envelope breakdown (Figure 1; Maciejowski et al., 2010; Rodriguez-Bravo et al., 2014; Liu et al., 2017). Following this, a Bub1-centered, attachment-independent timer is activated in a BubR1-PP2A-B56 (Protein Phosphatase 2A) dependent manner for transient interaction and recruitment of Mad1, serving as a rapid SAC activator during early mitosis (Figure 1; Qian et al., 2017). Recent discovery of Aurora A in sustaining Mad2 level at the kinetochores also adds a new dimension to the SAC-mediated control for the sensing of kinetochore-microtubule attachment (Figure 1; Courtheoux et al., 2018).

Prompt and efficient SAC silencing is essential for resumption of mitotic progression once proper microtubule-kinetochore attachment is achieved. However, the molecular controls underlying this inactivation process has remained poorly understood until recently. During the activation of SAC, phosphorylated KNL1 (outer kinetochore protein) interacts with Bub1 and Bub3 for subsequent kinetochore recruitment of BubR1, Cdc20, Mad1, and Mad2 (Shepperd et al., 2012; Primorac et al., 2013). Once all chromosomes are bi-oriented, progressive dampening of the checkpoint is initiated by the disassociation of checkpoint proteins from the kinetochores by dephosphorylation of KNL1 mediated by PP2A-B56 via binding of BubR1 and PP1 (Protein Phosphatase 1) recruited by KNL1 and the Ska (spindle/kinetochore-associated) complex (Figure 1; Rosenberg et al., 2011; Espert et al., 2014; Nijenhuis et al., 2014; Sivakumar et al., 2016). PP1 also dephosphorylates Mps1 and kinetochore-localized Cdc20, thus inhibiting Mps1 catalyzed phosphorylation of KNL1 and promoting APC/C activation, respectively (Figure 1; Kim et al., 2017; Moura et al., 2017). In addition to the dampening of SAC at kinetochores, TRIP13 and p31^comet^ promote the disassembly of free MCC by conformational inactivation of Mad2, thus preventing the formation of MCC-APC/C complex (Figure 1; Jia et al., 2011; Teichner et al., 2011; Wang K. et al., 2014; Miniowitz-Shemtov et al., 2015; Brulotte et al., 2017). Ubiquitination of Cdc20 and BubR1 further aids the release of APC/C from the MCC (Figure 1; Sitry-Shevah et al., 2018). The destabilization of BubR1 is a consequence of HDAC2/3 mediated deacetylation of BubR1 for which BRCA2 serves as a signaling platform (Figure 1; Park et al., 2017).

While SAC is essential in suppressing APC/C activity to halt mitotic exit, other regulators may also contribute as suggested by recent studies. The deubiquitinase USP9X modulates the stability of MCC:APC/C complex thereby influencing the SAC dynamics (Skowyra et al., 2018). The E2 ubiquitin-conjugating enzymes UBE2C, UBE2S, and UBE2D may impact the stability of MCC:APC/C complex and might also directly affect APC/C (Skowyra et al., 2018). This raises the possibility that cells with low APC/C activity can sustain mitotic arrest and prevent premature mitotic slippage (Wild et al., 2016). Oxidative stress induced by H_2_O_2_ is also able to arrest cells in mitosis independently of SAC in yeast (Atalay et al., 2017). How these elements are regulated to contribute to the fine tuning of mitotic exit-SAC axis is not entirely clear.

## The Death Pathway in Mitosis

A direct consequence of an exceedingly prolonged mitosis is the activation of the death pathway that resembles, in its characteristics, the intrinsic/mitochondrial pathway of programed cell death or apoptosis. Unlike the extrinsic apoptotic pathway, which is triggered first by activation of cell surface death receptor, formation of death-inducing signaling complex and subsequently through activation of caspase 8 and 10 dependent cascades (Dickens et al., 2012), the intrinsic apoptotic pathway is initiated by the activation of pro-apoptotic and the inactivation of anti-apoptotic proteins (predominantly from the Bcl-2 protein family). The activation of pro-apoptotic proteins signals the recruitment and oligomerization of the Bcl-2 effector proteins Bax (Bcl-2-associated X protein) and Bak (Bcl-2 antagonist or killer) on mitochondria. This leads to the mitochondrial outer membrane permeabilization (MOMP), triggering the release of pro-apoptotic mitochondrial intermembrane-space proteins such as cytochrome c, which in turn facilitates the formation of apoptosome [constituents: cytochrome c, Apaf-1 (apoptotic protease activating factor 1) and procaspase-9]. This results in the caspase-9 dependent activation of a caspase cascade and subsequently, nucleosome fragmentation, cytoskeleton deformation and phagocytosis (Lopez and Tait, 2015; Fox and MacFarlane, 2016).

**FIGURE 2:**
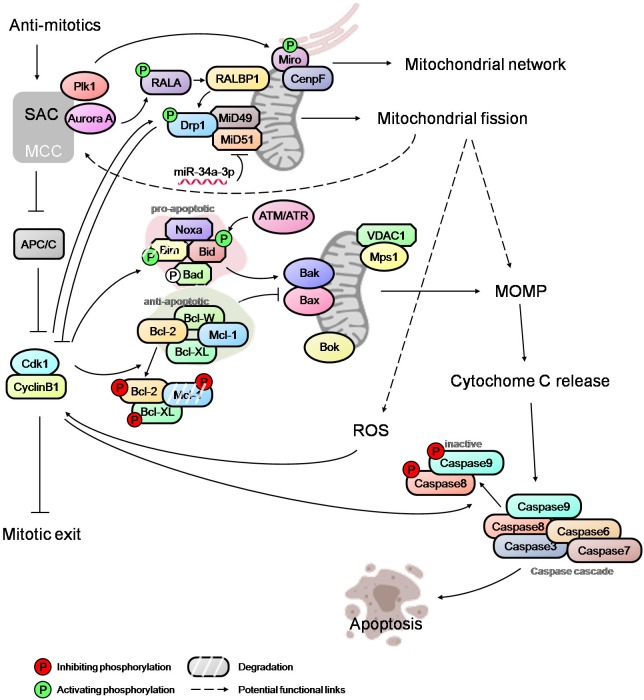
An overview of mitotic cell death network. Integration of SAC, mitochondrial fission, MOMP and caspase activation via regulatory links and potential cross-talks (dashed arrows). The modulators of SAC, such as Plk1 and Aurora A, regulate the mitochondrial network and mitochondrial fission during mitosis, and may affect the level of ROS and MOMP. Mitotic kinase Mps1 can also localize to mitochondria and potentially influence the process of cytochrome c release. High Cdk1-cyclinB1 level, caused by prolonged mitotic arrest, further activate/inactivate the pro-apoptotic and anti-apoptotic proteins, thus tipping the balance in favor of Bak/Bax oligomerization at mitochondria and, subsequently, MOMP. Opposing these cell death-conducive events are the death-suppressive effectors. Cdk1-cyclinB1 mediates the inactivation of caspases, potentially counteracting the pro-death signaling resulting from MOMP and cytochrome c release. The mitochondrial fission mediator Drp1 appears to antagonize Cdk1-cyclinB1by promoting slippage while the elevated ROS level correlates with higher Cdk1-cyclinB1 activity favoring a sustained mitotic arrest. Thus, the fate of a mitotically arrested cell is determined by the complex interactions between various regulatory modules.

Phosphorylation of anti-apoptotic proteins, such as Bcl-2, Bcl-XL and Mcl-1, during prolonged mitosis has been considered the priming event in mitotic cell death signaling. These phosphorylation events (mediated by Cdk1 and SAC proteins) act to disable the anti-apoptotic proteins and promote their degradation, thus tipping the balance in favor of pro-apoptotic signal (Figure 2; Harley et al., 2010; Terrano et al., 2010; Wang et al., 2011; Eichhorn et al., 2013). Another anti-apoptotic protein, Bcl-W, has also been reported to act as a negative regulator of mitotic cell death (Huang et al., 2016). Though phosphorylation of the anti-apoptotic proteins, which also occurs during normal mitosis, may prime the mitotically arrested cells to apoptosis, it is unlikely to trigger cell death without a multitude of subsequent events such as activation of Bax/Bak by pro-apoptotic proteins, priming of mitochondria for cytochrome C release, etc. The pro-apoptotic proteins, such as Bid, Bad and Bim, also undergo phosphorylation due to activation of multiple kinases in mitosis (Figure 2; Mac Fhearraigh and Mc Gee, 2011). While the ATM/ATR mediated phosphorylation of Bid promotes cell death in mitosis, phosphorylation of Bad in mitosis does not promote apoptosis (Berndtsson et al., 2005; Wang P. et al., 2014). The role of Bim in initiating apoptosis during mitotic arrest is debatable, with some reports showing that Bim phosphorylation is critical for the priming of cell death while others suggesting that Bim phosphorylation results in its proteolytic degradation (Moustafa-Kamal et al., 2013; Han et al., 2014; Wan et al., 2014; Haschka et al., 2015). Recent studies have implicated Noxa as an important apoptotic initiator in mitosis; however, the endogenous level of Noxa is very low or undetectable during mitosis (Moustafa-Kamal et al., 2013; Díaz-Martínez et al., 2014; Han et al., 2014; Wan et al., 2014; Haschka et al., 2015). It is noteworthy that Cdk1 also modulates the phosphorylation of caspase 8 and 9 resulting in the inactivation of the caspase cascade, thus shielding cells against mitotic death (Figure 2; Allan and Clarke, 2007; Matthess et al., 2010). How cells coordinate the priming of cell death, the balance between pro-apoptotic and anti-apoptotic proteins, and the death inhibitory signal due to caspase phosphorylation merit further investigation. Potential new apoptotic mediators such as Bok which can initiate MOMP independently of Bax, Bak and Bcl-2 family proteins (Llambi et al., 2016) also warrant attention.

## Mitochondrial Dynamics and Functions During Prolonged Mitosis

Many studies on mitotic cell death have focused extensively on the mitotic control of anti-apoptotic and pro-apoptotic proteins. However, these studies are incomplete without the understanding of the mechanistic modulation of the mitotic death signaling network as well as mitochondrial dynamics where the apoptotic signals eventually converge to cause MOMP and the release of death-promoting proteins (Díaz-Martínez et al., 2014; Kalkavan and Green, 2017). Mitochondria are vital organelles required for multiple fundamental cellular processes including ATP production through oxidative phosphorylation, generation/regulation of reactive oxygen species (ROS), maintenance of calcium and iron homeostasis, and the modulation of programed cell death (Osellame et al., 2012; Friedman and Nunnari, 2014). These functions of mitochondria are tightly linked to the dynamic changes in their morphology induced by the fusion/fission cycle during cell division. Mitochondria normally form an tubular network during interphase; however, they undergo extensive fission during mitosis. Regulation of mitochondrial fission is mediated by Drp1 (Dynamin-related protein 1) which is recruited by Fis1, Mff, MiD49 and MiD51 (mitochondrial dynamics proteins of 49 and 51 kDa) at the mitochondrial outer membrane, while fusion is mediated by mitofusins Mfn1 and Mfn2 (located at the outer mitochondrial membrane) and by Opa1 (located at the inner mitochondrial membrane). The network regulating mitochondrial dynamics has been reviewed extensively (Losón et al., 2013; Wai and Langer, 2016; Maycotte et al., 2017).

At the onset of mitosis, mitochondria undergo extensive fission due to RALA and RALBP1 mediated phosphorylation and the activation of Drp1 by Cdk1 (Figure 2; Taguchi et al., 2007; Kashatus et al., 2011). Recent studies have implicated the Drp1 adapter proteins, MiD49 and MiD51, which are epigenetically modulated by miR-34a-3p microRNA, in facilitating mitochondrial fission during mitosis (Figure 2; Chen et al., 2018). It has been suggested that mitochondrial fission promotes MOMP and sensitizes cells to apoptosis downstream of Bak/Bax activation (Suen et al., 2008; Brooks et al., 2011). However, more recent studies have shown that mitochondrial fission and fragmentation promotes apoptotic resistance by directly controlling Bak/Bax function in MOMP in that mitochondrial fragmentation restricts the Baxα9-mitochondria membrane interactions (Kashatus et al., 2015; Renault et al., 2015). Hence, the role of the mitochondrial fission in the modulation of mitotic cell death requires a careful examination.

While MOMP is critical for induction of cell death, weak MOMP with limited activation of caspase is not sufficient for inducing cell death and instead results in genomic instability and tumorigenesis (Ichim et al., 2015). This has raised the question whether tipping of the balance in favor of pro-apoptotic proteins during prolonged mitosis is sufficient to trigger cell death. Interestingly, an amino-terminally truncated isoform of Mcl-1, in addition to its role in sequestering anti-apoptotic proteins during interphase, can localize to mitochondrial matrix to facilitate mitochondrial functions (Perciavalle et al., 2012). Whether this Mcl-1 isoform affects mitochondrial functions such as ATP production, maintenance of mitochondrial fusion and cristae structures and if these effects do impinge on the regulation of mitotic cell death require further investigations.

## Mapping the Mitotic Cell Death Signaling Network

Anti-mitotic agents that alter spindle-kinetochore attachment dynamics lead to the activation of SAC, which then prevents both the onset of anaphase and mitotic exit, thus causing cells to arrest in prometaphase. The SAC accomplishes this via the inhibition of APC/C activity, thereby maintaining substantial levels of securin, cyclin B1, and Cdk1 activity. Cdk1, in turn, phosphorylates multiple pro-apoptotic/anti-apoptotic proteins and caspases, and mediates mitochondrial fission. It is known that cells are unable to maintain the high Cdk1 activity-status indefinitely due to slow erosion of cyclin B1 levels despite the active checkpoint (Brito and Rieder, 2006). Given these diverse regulations at play, cells can adopt two different fates following the checkpoint-imposed prolonged arrest: mitotic cell death or mitotic slippage. Substantial efforts have been made to map the complex signaling network regulating the mitotic cell death. Early studies had proposed a competing-networks model according to which the pathways mediating mitotic slippage (measured by the decline in cyclin B1 level) and the mitotic cell death (quantified by the pro-death signals) are mechanistically independent. The activity thresholds and stochastic competition between the two pathways dictate the final cell fates during an extended mitotic arrest (Gascoigne and Taylor, 2009; Huang et al., 2010). Based on the observation that the time span of mitosis does not correlate with cell fate, this model marks cyclin B-Cdk1 as the key element that controls both slippage and apoptosis (Gascoigne and Taylor, 2008; Brito and Rieder, 2009). This implies that the primary function of SAC is to restrain APC/C activity and maintain sufficient cyclin B-Cdk1 levels instead of directly signaling to apoptotic pathway. Since strategies to augment and stabilize cyclin B-Cdk1 (other than inhibiting APC/C activity) are limited, targeting cytokinesis may be a feasible alternative to prevent mitotic exit. With this notion in view, a new strategy that inhibits cytokinesis independently of SAC, for instance by targeting PRC1 (protein regulator of cytokinesis 1), has been used to enhance the lethal effects of anti-mitotic agents (Liu et al., 2018).

However, recent evidence suggests a possible link between the SAC network and the mitochondrial dynamics/functions that may potentially regulate the death signaling. The SAC protein Mps1 improves cells’ viability during mitosis by translocating to mitochondria and binding to VDAC1 (voltage-dependent anion channel 1), raising the possibility of a role for Mps1 in the regulation of cytochrome c release (Figure 2; Zhang et al., 2016). Another SAC regulator, Plk1, is reported to phosphorylate the mitochondrial Rho GTPase Miro, leading to mitochondrial morphology changes, Ca^2+^overloading in mitochondria and an apoptotic response in neural stem cells (Figure 2; Lee et al., 2016). Miro also recruits CENP-F to mitochondria to help modulate the mitochondrial network and promotes mitochondrial redistribution during mitosis (Figure 2; Kanfer et al., 2015). Given that multiple signaling networks converge onto mitochondria in addition to the well-established pro- and anti-apoptotic players, an in-depth understanding of the regulatory relationship between mitochondrial dynamics/functions, mitotic machinery and mitotic checkpoint controls is essential for unraveling the complexity of the mitotic cell death network. Such a network may potentially include feedback mechanisms originating from mitochondria regulatory machinery or the apoptotic signaling modules and converging onto the checkpoint control. A genome-wide siRNA screen has indicated a potential role for Drp1 in promoting mitotic slippage and in generating ROS via mitochondrial fragmentation. This appears to correlate with an increase in the cyclin B1 level (Figure 2; Daum et al., 2011; Díaz-Martínez et al., 2014; Gupte, 2015).

While new evidence is emerging to help further dissection of SAC silencing, it is useful to consider therapeutic benefits of impairing SAC silencing to enhance mitotic cell death. Disruption of SAC-silencing would not only prolong mitotic arrest and enhance MOMP, but may also engender other cellular disparities, such as cohesion fatigue and cell fate alterations, that are detrimental to the survival of the mitotically arrested tumor cells (Rieder and Maiato, 2004; Daum et al., 2011; Gorbsky, 2013; Pilaz et al., 2016). Hence, targeting the SAC silencing machinery may provide a new strategy for the development of the next generation of anti-mitotic agents for combinatorial therapies.

## Conclusion

The mitotic cell death is governed by a complex network that coordinates SAC activation and silencing, levels of cyclin B1-Cdk1 activity, balance between pro- and anti-apoptotic proteins, extent of MOMP, mitochondrial morphology and caspase activation. A deeper knowledge of how the mitochondrial dynamics/functions, the death signaling and the mitotic checkpoint controls are integrated in the context of prolonged mitosis may provide a new perspective on the regulatory underpinning of the cell death network and highlight potential therapeutic strategies for combating chemo-resistance and genome instability induced by anti-mitotic agents.

## Author Contributions

WR wrote some sections of the manuscript. HL wrote some parts of the manuscript. US wrote and edited the manuscript.

## Conflict of Interest Statement

The authors declare that the research was conducted in the absence of any commercial or financial relationships that could be construed as a potential conflict of interest.
